# Congenital Malaria in China

**DOI:** 10.1371/journal.pntd.0002622

**Published:** 2014-03-13

**Authors:** Zhi-yong Tao, Qiang Fang, Xue Liu, Richard Culleton, Li Tao, Hui Xia, Qi Gao

**Affiliations:** 1 Department of Parasitology, Bengbu Medical College, Bengbu, People's Republic of China; 2 Bengbu First People's Hospital, Bengbu, People's Republic of China; 3 Malaria Unit, Institute of Tropical Medicine, Nagasaki University, Sakamoto, Nagasaki, Japan; 4 Jiangsu Institute of Parasitic Diseases, Wuxi, People's Republic of China; 5 Key Laboratory on Technology for Parasitic Disease Prevention and Control, Ministry of Health, Wuxi, People's Republic of China; Institute of Tropical Medicine (NEKKEN), Japan

## Abstract

**Abstract:**

**Background:**

Congenital malaria, in which infants are directly infected with malaria parasites from their mother prior to or during birth, is a potentially life-threatening condition that occurs at relatively low rates in malaria-endemic regions. It is recognized as a serious problem in *Plasmodium falciparum*–endemic sub-Saharan Africa, where recent data suggests that it is more common than previously believed. In such regions where malaria transmission is high, neonates may be protected from disease caused by congenital malaria through the transfer of maternal antibodies against the parasite. However, in low *P. vivax*–endemic regions, immunity to vivax malaria is low; thus, there is the likelihood that congenital vivax malaria poses a more significant threat to newborn health. Malaria had previously been a major parasitic disease in China, and congenital malaria case reports in Chinese offer valuable information for understanding the risks posed by congenital malaria to neonatal health. As most of the literature documenting congenital malaria cases in China are written in Chinese and therefore are not easily accessible to the global malaria research community, we have undertaken an extensive review of the Chinese literature on this subject.

**Methods/Principal Findings:**

Here, we reviewed congenital malaria cases from three major searchable Chinese journal databases, concentrating on data from 1915 through 2011. Following extensive screening, a total of 104 cases of congenital malaria were identified. These cases were distributed mainly in the eastern, central, and southern regions of China, as well as in the low-lying region of southwest China. The dominant species was *P. vivax* (92.50%), reflecting the malaria parasite species distribution in China. The leading clinical presentation was fever, and other clinical presentations were anaemia, jaundice, paleness, diarrhoea, vomiting, and general weakness. With the exception of two cases, all patients were cured with antimalarial drugs such as chloroquine, quinine, artemether, and artesunate.

**Conclusions:**

The symptoms of congenital malaria vary significantly between cases, so clear and early diagnosis is difficult. We suggest that active surveillance might be necessary for neonates born to mothers with a history of malaria.

## Introduction

Malaria is a mosquito-borne infectious parasitic disease that is prevalent in tropical and subtropical areas. It is estimated that 660,000 lives were lost to the disease in 2010, mostly in sub-Saharan Africa, a region hyperendemic for *P. falciparum*, the most virulent of the species that cause malaria in humans. Over 86% of malarial deaths in this area were of children under 5 years of age [1], [2]. In China, *P. vivax* is the most prevalent species of malaria parasite [3]. Following a dedicated malaria control effort, the incidence of malaria in China has been reduced to just 0.0334 cases per 10,000 people (data from 2011) [4]. In 2010, a national elimination programme, the “Action Plan of China Malaria Elimination (2010–2020),” was initiated [5], [6]. Despite this, there are continued outbreaks of *P. vivax* malaria in the central region of the country, where unstable transmission of the parasite remains [7]. Congenital malaria due to *P. falciparum* is reportedly rare in hyperendemic areas, where pregnant woman can pass on antibodies against the parasite to their newborn babies. Thus, even though maternal asymptomatic malaria is common, congenital malaria is not [8]–[11]. However, recent reports suggest that congenital *P. falciparum* malaria is not as rare as previously believed [12]–[16]. Whilst there are differing opinions regarding the incidences of congenital malaria, the effects on neonates of this prenatal disease are clear [17]. In low *P. vivax*-endemic regions, immunity to vivax malaria is also low, and, thus, there is the possibility that congenital vivax malaria poses a significant threat to the health of the foetus [18]. During the long battle against malaria, numerous congenital malaria cases were reported in the Chinese medical literature. However, because of language barriers, this valuable information has not been accessible to the malaria research community outside of China. Here, we review congenital malaria cases from three major publicly searchable Chinese databases, concentrating on data from 1915 through 2011 [19], [20].

## Methods

We examined all published cases of congenital malaria between 1915 and May 2011 in the Chinese literature electronically available in the CNKI (China National Knowledge Infrastructure, www.cnki.net), Wanfang Data (www.wanfangdata.com), and CSTJ (China Science and Technology Journal Database, www.cqvip.com) databases [20]. The keywords for the literature search strategy were “congenital OR neonatal AND malaria”. All pertinent case reports were downloaded and analysed in the context of the epidemiology of the disease in China. The criteria used to diagnose congenital malaria were: (1) whether the time of the neonate's onset of malaria symptoms was within 7 days of delivery; (2) in malaria nonendemic areas, if the onset of symptoms was within 30 days of birth; (3) in malaria endemic areas, if the onset of symptoms was within 30 days of delivery (with a corresponding diagnosis of malaria in the mother at the time of birth); and (4) if the onset of both the neonate's and the mother's symptoms and the time of delivery were during the nontransmission season (and therefore due to relapse caused by activated hypnozoites in the mother). Cases of congenital malaria reported in English were also included [21]. SPSS 14.0 (SPSS Inc., United States) was used for statistical analysis.

## Results

### Temporal and spatial distribution of congenital malaria

**Figure 1:**
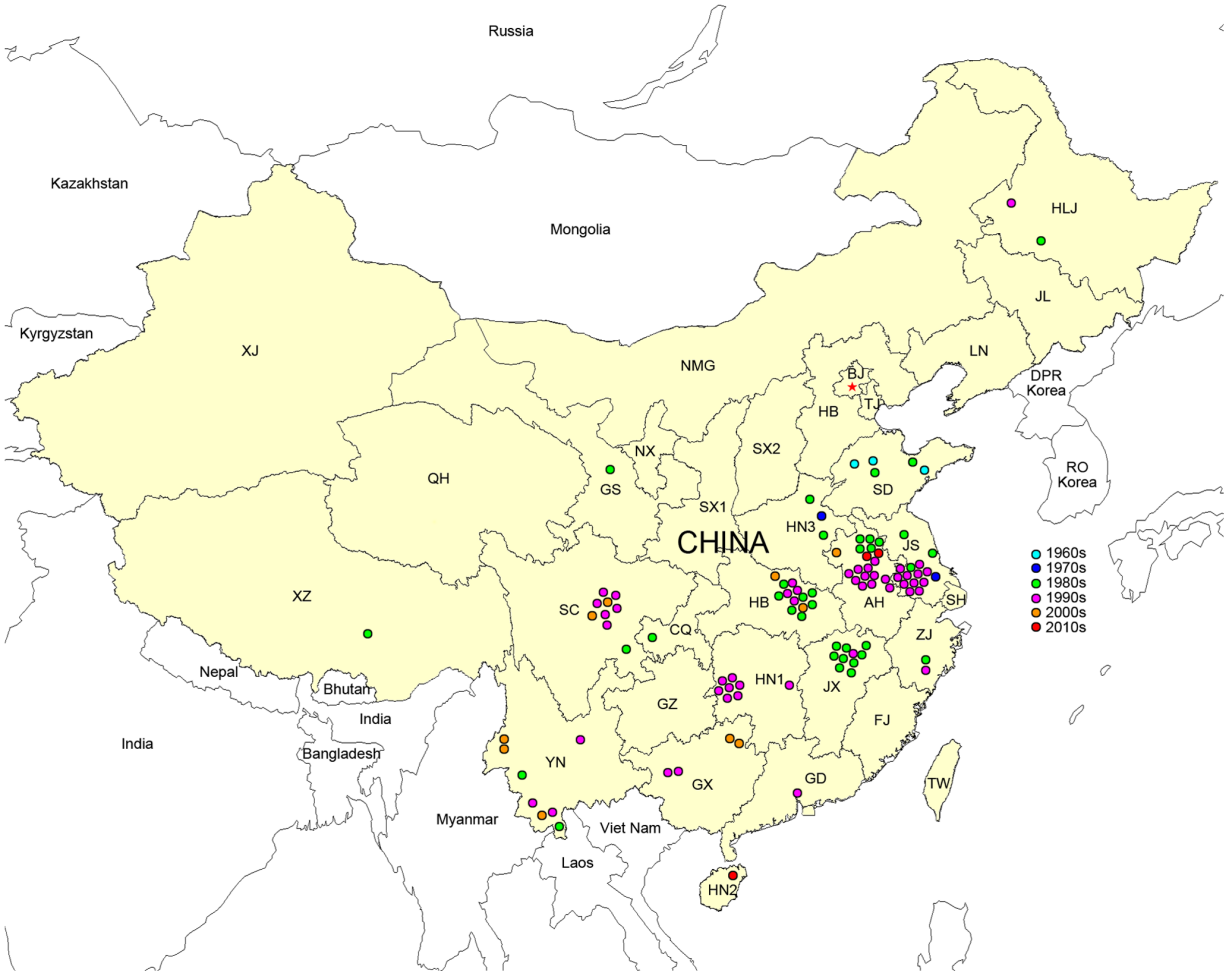
Congenital malaria in China from 1963 to 2010. Abbreviations of affected provinces: AH: Anhui; CQ: Chongqin; GS: Gansu; GX: Guangxi; GD: Guangdong; HB: Hubei; HLJ: Heilongjiang; HN1: Hunan; HN2: Hainan; HN3: Henan; JS: Jiangsu; JX: Jiangxi; SC: Sichuan; SD: Shandong; XZ: Xizang; YN: Yunnan; ZJ: Zhejiang.

In this review, through the collection and interpretation of data originating between 1915 and 2011 and deposited in electronic periodical databases, there were 103 congenital malaria cases reported by 61 medical articles in Chinese and one case occurring in mainland China reported in English [21] that met the requirements of the diagnostic criteria described above. The earliest report dates from 1963 and the latest was from 2010. The majority of the reports (81.73%) were published in the 1980s and 1990s. Congenital malaria mainly occurred in the eastern, central, and southern regions of China, and also in the low-elevation area in southwest China (cases in these areas account for 95.19%). The provinces with the highest incidences were the Anhui (19 cases, 18.29%), Jiangsu (15 cases, 14.42%), and Hubei (13 cases, 12.50%) provinces in the river valley sections of the Yangtze and Huaihe River (Figure 1).

### Laboratory diagnosis

Of the 104 cases of congenital malaria, 80 clearly identified the species of malaria parasite involved. The predominant species was *P. vivax* (74 cases, 92.50%), followed by *P. falciparum* (five cases, 6.25%) and *P. malariae* (one case, 1.25%). All diagnoses were performed by microscopy. In 85 cases, parasites were found initially in peripheral blood films, while in nine cases, parasites were initially detected in bone marrow smears. For the remaining ten case reports, there were no descriptions of which material had been used for diagnosis.

### Case presentation

**Figure 2:**
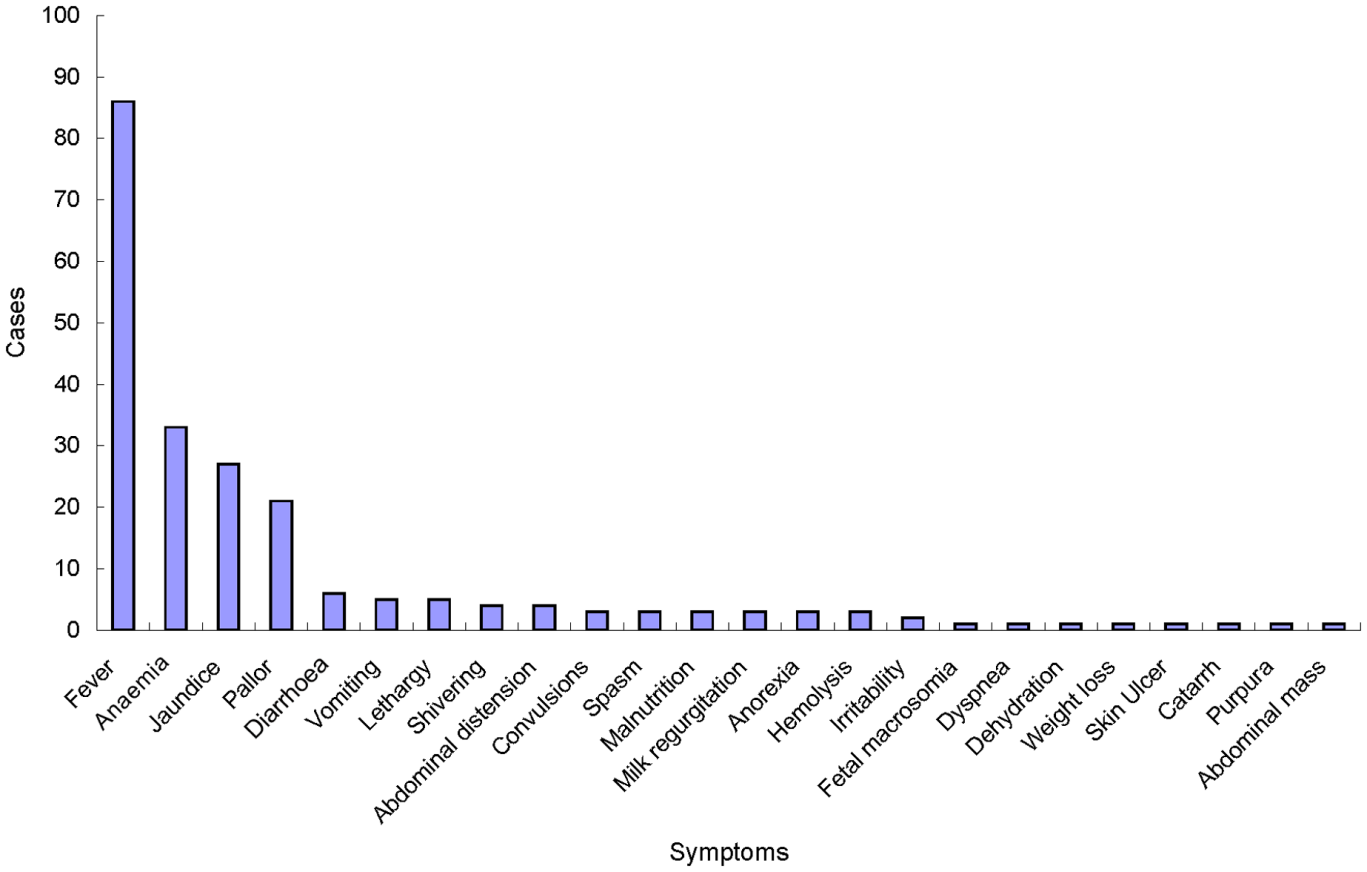
Symptoms of 96 infants with congenital malaria.

Of the 104 cases of congenital malaria, 96 reported clinical symptoms (Figure 2), of which 86 (89.58%) described fever. In this fever group, 78 had fever as the main presentation; only 27 described intermittent fever. The other clinical presentations were anaemia (33 cases, 34.38%), jaundice (27 cases, 28.13%), paleness (21 cases, 21.88%), diarrhoea (six cases, 6.25%), vomiting (five cases, 5.21%), weakness and inactivity (five cases, 5.21%), shivers (four cases, 4.17%), and abdominal distention (four cases, 4.17%). Among the 60 cases of congenital malaria in which liver palpation results were described, 57 cases had a liver that was palpable below the right costal margin. Of the 51 cases in which the enlarged liver was measured, 41 infants had livers enlarged with a span of ≥2 cm below the right costal margin. In addition, four infants were reported to be hepatomegalous, but the exact measurements were not given. Spleen palpations were recorded in 63 cases. Of these, 54 cases were palpable below the left costal margin. 40 cases of enlarged spleen were measured, of which 35 cases presented enlargement ≥2 cm; an additional nine cases were described only as splenomegaly.

### Laboratory examination

Of 59 infants with congenital malaria for whom haemoglobin concentrations (Hb) were recorded, 51 had Hb<120 g/L, 43 had Hb<90 g/L, 26 had Hb<60 g/L and four had Hb<30 g/L. Thirteen of the 20 infants with congenital malaria for whom BPCs (blood platelet counts) were performed had thrombocytopenia (PLTs [platelets])<100×10^9^/L. Of 53 infants with congenital malaria for whom WBC (white blood cell) counts were performed, ten infants had WBC>12.5×10^9^/L.

### Diagnosis

The 88 cases of congenital malaria in newborns whose ages at the onset of symptoms were recorded ranged from 1 to 50 days (median, 8 days). In those cases in which there was a clear diagnosis of malaria (*n* = 67), the age distribution was 4 to 126 days (median, 30 days). The time interval from the symptom onset to diagnosis ranged from 1 to 111 days (median, 15 days).

### Maternal history

Of 86 women with maternal history documented and newborns diagnosed as having congenital malaria, all had at least one record of malaria. In five cases, the malaria occurred during their first trimester of pregnancy, 20 cases had malaria in the second trimester, 17 during their third trimester, 46 during prenatal, and four cases were unspecified. Eight women had recorded malaria cases prior to their pregnancy (five of which occurred only before their pregnancy). Three women had no recorded malaria history.

### Treatment and results

**Figure 3:**
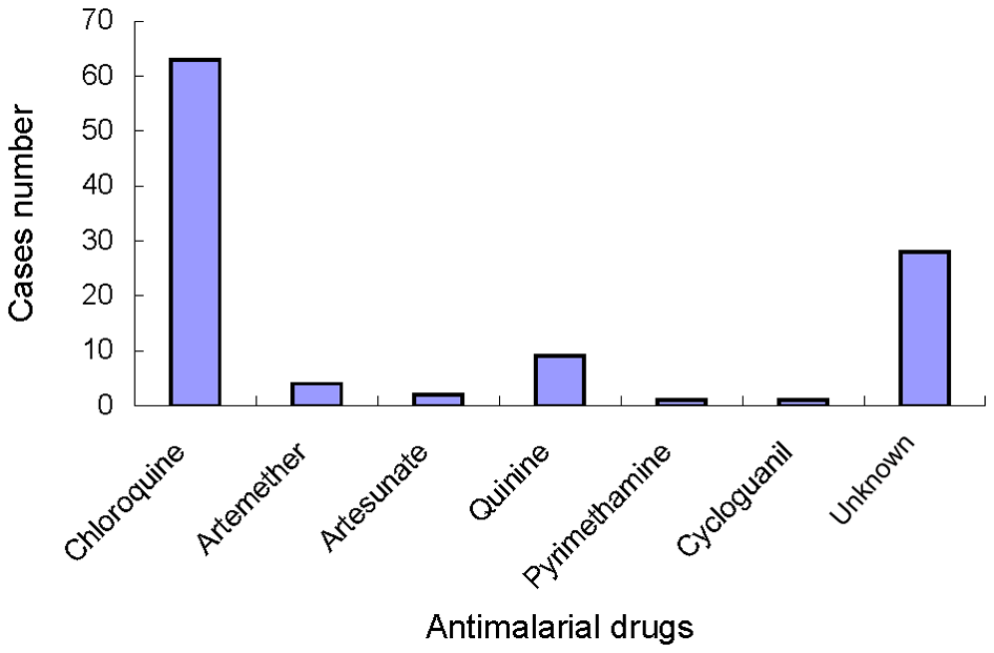
Antimalarial drug usage in 102 cases of congenital malaria.

Of the 104 infants with congenital malaria, 102 were cured with one or two types of drugs after receiving antimalarial treatment (Figure 3). Sixty-three were treated with chloroquine, four were treated with artemether, two were treated with artesunate, nine were treated with quinine, one was treated with pyrimethamine, one was treated with cycloguanil, and 28 were treated with unspecified antimalarial drug(s). In addition, nine infants received primaquine along with an antimalarial drug related to those described above. However, one infant's family refused and halted treatment, and another infant was too ill and died two hours after diagnosis.

## Discussion

Congenital malaria is defined as the infection of an infant with malaria parasites from the mother during pregnancy or during birth and not from the bite of an infected mosquito or through blood transfusion. The transmission of parasites may be through placental passage during pregnancy or as the baby passes through the birth canal during labour. According to Chinese medical literature surveyed for this review, congenital malaria in China was (and is) relatively rare.

China has previously been hyperendemic for malaria. In the peak years of 1960 and 1970, malaria cases reached up to 30 million and 23 million, respectively [22]–[24]. Prior to the appearance of SARS (severe acute respiratory syndrome) in 2003, there was no effective system for collecting information concerning epidemics and disseminating it to the public [25], [26]. It is therefore difficult to unearth accurate statistics concerning malaria cases, including congenital malaria. However, currently available electronic medical literature databases have provided us with an overview of the diagnosis and treatment of congenital malaria occurring in China from 1963 to 2011. During this time period, there were 104 cases of congenital malaria reported in the Chinese (103) and English (1) literature. For the 80 cases in which the causal malaria parasite species was identified, 74 (92.50%) cases were caused by *P. vivax*, which is in accordance with the fact that this species was the most prevalent in China during this period. In this review, 95.19% cases were from east, middle-south, and southwest China in the Yangtze and Huaihe river basins, which correlates with the reported malaria epidemic areas in China [27]–[29]. Although China's malaria hyperendemic period occurred in the 1960s and 1970s, cases of congenital malaria were more frequently reported in the 1980s and 1990s. This is possibly due to the fact that periodicals were routinely deposited in databases after 1979. Prior to this time, China's social and economic development did not afford accurate reporting of congenital malaria cases [30].

It has been previously assumed that the onset of congenital malaria symptoms in the neonate occurs between 10 and 30 days. However, in this review, we found that the age at symptom onset was somewhat younger (median 8 days). One reason for this difference may be that our diagnosis criteria were more conservative than those used in many previous studies. For example, if an infant was older than 30 days at the onset of malaria symptoms, we stipulated that both the time of birth and the onset of symptoms should have occurred during a nontransmission season in order to be considered as a congenital malaria case. Upon review, some cases did not meet the classification requirements of congenital malaria, while other cases were reclassified from neonatal malaria to congenital malaria.

We found that the most prominent symptom of congenital malaria was fever (89.58%, 86/96 cases). Other common symptoms included anaemia (34.38%, 33/96 cases), jaundice (28.13%, 27/96 cases), and paleness (21.88%, 21/96 cases). Most cases of neonatal malaria presented with fevers that did not closely resemble typical malaria paroxysms. For instance, 27 congenital malaria neonates had intermittent fever (28.13%, 27/96 cases), but only four cases had shivering (4.17%, 4/96 cases), and a further ten cases of neonatal malaria were reported as afebrile (10.42%, 10/96). Hepatomegaly and splenomegaly have previously been reported as common during congenital malaria. However, the criteria for the diagnosis of hepatosplenomegaly are not consistent. We found that 80.39% (41/51 cases) of the infants' livers were ≥2 cm below the right costal margin, and 87.50% (35/40 cases) of the infants' spleens were enlarged ≥2 cm below the left costal margin.

There was a considerable delay in the time taken to diagnose congenital malaria; the time interval from initial symptom onset to diagnosis of congenital malaria was 1–111 days (median 15 days). There are several possible reasons for this delay. First, it is possible that the initial symptoms of congenital malaria were not clear enough for a confident diagnosis near their onset. Second, although routine blood tests were mandatory for hospitalized patients in China, malaria screening is not routinely ordered. Third, although the automatic haematology analyser has been widely used for blood screening in laboratories, they often failed to find the parasite [31], and blood film examination, which is crucial for malaria diagnosis, was often not performed. Finally, there is a lack of laboratory diagnostic methods such as RDT (rapid diagnostic test) or PCR (polymerase chain reaction) [32], [33]. All cases of congenital malaria were confirmed by microscopy of blood or bone marrow smears. RDT- or PCR-based diagnoses were not performed in any of the cases reported. According to WHO (World Health Organization) guidelines, microscopy or RDT should be used for a patient who is assumed to have had malaria before administering antimalarial treatment [1]. RDT has many proven benefits for malaria diagnosis in the field. However, the use of RDT in China is still relatively uncommon [34]. PCR is another diagnostic tool for malaria parasite identification and also plays an important role in the diagnosis of submicroscopic malaria. In order to overcome the problems associated with delayed diagnosis of congenital malaria, RDT and PCR methods should be introduced into clinical malaria management and malaria control programmes in conjunction with microscopy. Furthermore, improving the function of the automatic analysers used in routine malaria blood screening could lead to improvements in diagnosis by laboratory technicians and paediatricians [35].

Obviously, congenital malaria of the neonate is intrinsically dependent on the malaria status of the mother. Only three mothers of neonates diagnosed with congenital malaria reported no malaria symptoms, and all other mothers (96.51%, 83/86 cases) had malaria symptoms during or prior to pregnancy. Pregnant women are possibly more susceptible to malaria than nonpregnant woman, especially during first and second pregnancies [36], [37]. Due to the “One-Child Policy” implemented in China, most Chinese couples have only one or two children [38], so most pregnant women are either primigravid or secundigravid. This situation may create more chances for congenital malaria.

Both congenital and maternal malaria have impacts on the foetus, sometimes resulting in preterm deliveries and still births [17]. In the past, Chinese health care professionals may not have been vigilant enough in their assessment and diagnosis of malaria in their pregnant patients. This includes assessment and diagnosis of the resulting congenital malaria that may occur in neonates born to malarious mothers. Active surveillance for congenital malaria in infants whose mothers have been diagnosed as having malaria before labour began has not been noted or discussed in the literature. Even if a mother's malaria occurred prelabour, the infant was rarely screened for malaria. There is obviously a need in the future for heightened observance of malaria screening in newborns born to mothers with maternal malaria. Unfortunately, some health care providers neglect to treat expectant mothers due to an incorrect assumption that the treatment would increase the risk of adverse pregnancy outcomes. We found reports of 11 congenital malaria patients who did not receive any antimalarial treatment because both the patients and the doctors were concerned that antimalarial drugs were harmful to their newborns.

Malaria is usually transmitted through the bites of infected *Anopheles* mosquitoes; however, two cases of congenital malaria in this review were reported to have been associated with mothers who received blood transfusions during their labour. The donor's blood contained the malaria parasite, therefore infecting both mother and newborn. This issue suggests that blood transfusion history is an important factor to consider for malaria diagnosis from nonendemic areas.

Of 104 cases of congenital malaria analysed in this work, 102 were cured with antimalarial and symptomatic treatment; the main antimalarial drug was chloroquine (61.76%, 63/102 cases), effective due to the predominance of *P. vivax* in China. In addition, one infant died before antimalarial treatment commenced, and another infant was discharged from the hospital because the family refused treatment. As congenital malaria involves the direct transmission of the parasite from the blood of the mother to the blood of the foetus, there is no opportunity for hypnozoites to form in the liver of the infected neonate. As a consequence, congenital malaria caused by *P. vivax* may be treated solely with an antischizontal drug such as chloroquine, and antihepatic stage drugs are not required. However, nine congenital malaria case reviewed here received treatment with primaquine, a drug commonly used to treat hepatic stage parasites. This suggests that the clinician involved lacked sufficient understanding of the mechanism of congenital malaria and ignored the potential risk of side effects due to primaquine treatment.

The outcome of congenital malaria can be affected by factors such as the infecting species or the area in which it occurs [39]. Within the cases reviewed for this work, there was one reported fatality as a result of congenital malaria. In this case, maternal malaria was diagnosed during labour, and the newborn showed malarial symptoms shortly after the birth, but medical intervention was not initiated until the infant reached the age of 1 month. Unfortunately, the infant died of severe anaemia (Hb 22 g/L) at 40 days old (2 hours after being diagnosed as having malaria). In addition, in the high altitude area of China (Lhasa), another infant infected with *P. vivax* malaria had an episode of apnoea during the onset of malaria symptoms but survived. This suggests that congenital malaria may cause poor outcomes if prompt diagnosis and appropriate treatment are not received.

### Conclusions

As China is a country with a large population spread over a vast geographical area, health care and economic and social development levels differ greatly, especially between urban and rural areas. Usually, malaria-endemic areas in China occur in underdeveloped and more remote rural areas with very limited resources; these facts mean that medical personnel should pay more attention to the possibility of congenital malaria in order to ensure children's health and well-being.

**Key Learning Points**

- In low *P. vivax*–endemic regions, immunity to vivax malaria is also low; thus, there is the possibility that congenital vivax malaria poses a significant threat to the health of the foetus.
- We found that the most prominent symptom of congenital malaria was fever. Most cases of neonatal malaria presented with fevers that did not closely resemble typical malaria paroxysms.
- In China, many clinicians involved lacked sufficient understanding of the mechanism of congenital malaria and ignored the potential risk of side effects due to primaquine treatment.
- Active surveillance for congenital malaria in infants whose mothers have been diagnosed as having malaria before labour began has not been noted or discussed in the literature. Even if a mother's malaria occurred prelabour, the infant was rarely screened for malaria. There is obviously a need in the future for heightened vigilance of malaria screening in newborns born to mothers with malaria.

**Top Five Papers**

- Li T, He S, Zhao H, Zhao G, Zhu XQ (2010) Major trends in human parasitic diseases in China. Trends Parasitol 26: 264–270.
- World Health Organization (2012) Action Plan of China Malaria Elimination (2010–2020). Available: http://whothailand.healthrepository.org/handle/123456789/1460. Accessed 31 May 2013.
- Piñeros-Jiménez JG, Álvarez G, Tobón A, Arboleda M, Carrero S, et al. (2011) Congenital malaria in Urabá, Colombia. Malar J 10: 239.
- Liu X, Tao ZY, Fang Q, Wang XM, Zhang H, et al. (2012) A case of congenital *plasmodium vivax* malaria from a temperate region in Central China. Malar J 11: 182.
- Lesko CR, Arguin PM, Newman RD (2007) Congenital malaria in the United States: A review of cases from 1966 to 2005. Arch Pediatr Adolesc Med 161: 1062–67.
